# Elevated Temperature Induced Adaptive Responses of Two Lupine Species at Early Seedling Phase

**DOI:** 10.3390/plants10061091

**Published:** 2021-05-29

**Authors:** Sigita Jurkonienė, Jurga Jankauskienė, Rima Mockevičiūtė, Virgilija Gavelienė, Elžbieta Jankovska-Bortkevič, Iskren Sergiev, Dessislava Todorova, Nijolė Anisimovienė

**Affiliations:** 1 Nature Research Centre, Institute of Botany, Akademijos Str. 2, 08412 Vilnius, Lithuania; jurga.jankauskiene@gamtc.lt (J.J.); rima.mockeviciute@gamtc.lt (R.M.); virgilija.gaveliene@gamtc.lt (V.G.); elzbieta.jankovska@gamtc.lt (E.J.-B.); n.anisimoviene@gmail.com (N.A.); 2 Institute of Plant Physiology and Genetics Bulgarian Academy of Sciences, Acad. G. Bonchev Street, Bldg. 21, 1113 Sofia, Bulgaria; iskren@bio21.bas.bg (I.S.); dessita@bio21.bas.bg (D.T.)

**Keywords:** early growth stage, ethylene, IAA conjugates, indole-3-acetic acid, invasiveness, lupine seedlings, simulated conditions, warming simulation

## Abstract

This study aimed to investigate the impact of climate warming on hormonal traits of invasive and non-invasive plants at the early developmental stage. Two different lupine species—invasive *Lupinus polyphyllus* Lindl. and non-invasive *Lupinus luteus* L.—were used in this study. Plants were grown in climate chambers under optimal (25 °C) and simulated climate warming conditions (30 °C). The content of phytohormone indole-3-acetic acid (IAA), ethylene production and the adaptive growth of both species were studied in four-day-old seedlings. A higher content of total IAA, especially of IAA-amides and transportable IAA, as well as higher ethylene emission, was determined to be characteristic for invasive lupine both under optimal and simulated warming conditions. It should be noted that IAA-L-alanine was detected entirely in the invasive plants under both growth temperatures. Further, the ethylene emission values increased significantly in invasive lupine hypocotyls under 30 °C. Invasive plants showed plasticity in their response by reducing growth in a timely manner and adapting to the rise in temperature. Based on the data of the current study, it can be suggested that the invasiveness of both species may be altered under climate warming conditions.

## 1. Introduction

One of the most cited indicators of global climate change is the increase in global temperature. The impact of this factor on various plant growth and development processes has already been comprehended [1,2,3,4]. It is suggested that different non-native—alien—plant species may respond differently to the same climate changes and become invasive species—a threat to native species and biodiversity [2,3]. Model studies to date indicate that elevated temperature may increase invasion risk by accelerating physiological processes of alien species [5,6,7]. Therefore, the expected global warming with a predicted 5 °C rise during the 21st century may lead alien plant species to become invaders [1,2,5,8]. However, there is also evidence that the growth of plants may be adversely affected by temperature stress caused by warming [9]. These contradictory data on the global warming issue encourage us to study the adaptive response of alien plants to the rise in temperature by 5 °C.

Research on the molecular mechanisms determining the invasiveness of plants has only just begun [10,11]. The knowledge of plant physiological responses to climate warming will help to mitigate the impact of future environmental conditions on plants. Currently, there is no consensus among researchers regarding physiological-biochemical traits of plants that could determine invasiveness [10,11,12,13]. A special role of phytohormones in regulating invasiveness should be considered due to their involvement in plant growth, development and processes of response to environmental factors [14,15,16,17,18].

The role of indole-3-acetic acid (IAA) as a principal phytohormone coordinating developmental processes in response to environmental signals has been recognized [17,19,20,21,22]. Several studies have shown plant phenotypic adaptations of elongated hypocotyls and activation of IAA biosynthesis in certain plant tissues under elevated temperature [23,24]. It is suggested that growth responses to such conditions are partially regulated through the phytohormone IAA signaling pathway [25,26,27]. It is thought that the distribution of IAA and regulation of its content in cells and tissues could be important for the modulation of plant high-temperature response [25,28,29]. A direct connection between the IAA pathway and high temperature-induced adaptive growth has been shown in *Arabidopsis* hypocotyls [26,27]. However, in contrast, high temperature reduced IAA levels in the reproductive tissues of *Arabidopsis*, barley [25,28] and pepper [30]. Elkinawy [31] employed the method for cotyledon excision from four-day-old *Lupinus albus* intact seedlings and experimentally demonstrated the impact of IAA from cotyledons of lupines on the content of IAA in axial organs (hypocotyls). Additionally, it was stated that IAA synthesis de novo occurs in storage tissues (cotyledons) of the seeds during germination, post-germination and early developmental stages, mostly. A small amount of IAA can be synthesized in axial organs (hypocotyls) four days after seed swelling [32,33]. Thus, IAA level and turnover should be analyzed both in cotyledons and hypocotyls. The plant hormone ethylene has also been defined as a modulator of plant growth and development [34,35,36], though this is not its only role. Studies have shown that it enables plants to adapt to elevated temperature [18,37,38]. Increased ethylene emission reduced the growth of plants until the high temperature was removed [16,39,40].

Meanwhile, climate warming-induced adaptive responses of invasive plants are still not well studied. It is unclear how they depend on the IAA state and content in tissues and ethylene emission. The discovery of these characteristics may be carried out using a comparative study of genetically related plants [41,42,43,44]. Young intact lupine seedlings can be suitable objects to test the impact of increased temperature on IAA content and ethylene emission [44,45]. Such a model can provide a new insight into the hormonal traits of plant invasiveness onset detection and analysis under future climate warming scenarios. Thus, two different lupine species were tested in the current study.

We hypothesized that climate warming may affect the plant hormone regulatory system—the factor determining plant growth and development. Thus, the goal of the current study was to evaluate the adaptive responses of two lupine species under simulated climate warming conditions at the early phase of growth.

## 2. Results

### 2.1. Germination and Growth under 25 °C and 30 °C

**Figure 1:**
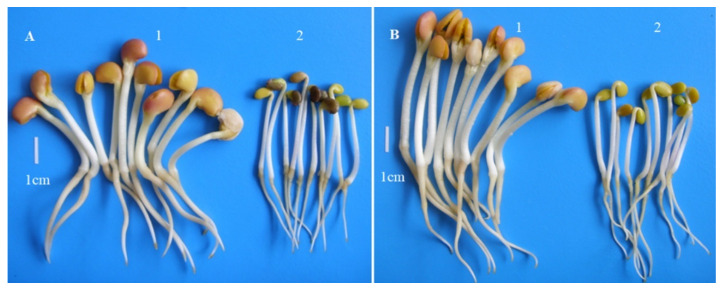
Four-day-old seedlings of non-invasive (*L. luteus*) (1) and invasive (*L. polyphyllus*) lupines (2) grown at 25 °C (**A**) and 30 °C (**B**). Scale bar, 1 cm.

The simulated climate warming conditions were found to differently affect seed germination and resulted in diverse growth responses of invasive and non-invasive lupines (Figure 1, Table 1). The data of the current study show that simulated 5 °C warming conditions had no significant effect on the seed germination percentage of non-invasive lupine. However, the seed germination of invasive lupine was 5% lower under 30 °C than under optimal conditions. The growth of non-invasive lupine was more intensive under 30 °C than under 25 °C. The weight of hypocotyls increased by 30%. The weight of roots and cotyledons was higher as well. On the other hand, elevated temperature (30 °C) resulted in slower growth of invasive lupine. The weight of hypocotyls and roots was about 40% and of cotyledons about 9% lower than that of plants grown under 25 °C (Figure 1, Table 1).

### 2.2. IAA Content under 25 °C and 30 °C

**Figure 2:**
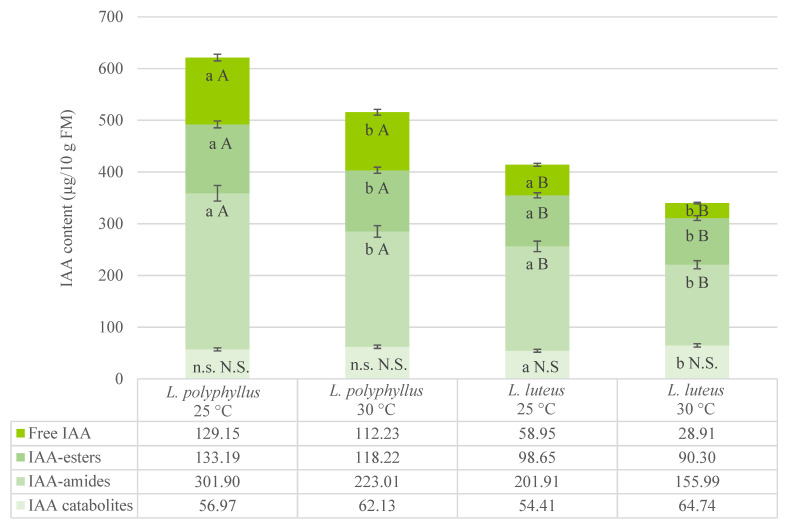
IAA content in cotyledons of seedlings grown at 25 °C and 30 °C. Vertical bars represent the total IAA content. Sub-bars depict the content of IAA compounds. Values are mean ± SD of three experiments with five replicates in each. Different uppercase letters indicate significant difference (*p* < 0.05) between mean values of two lupine species grown under the same temperature; N.S.—non-significant difference. Significant difference between mean values at 25 °C and 30 °C for each lupine species is marked with different lowercase letters; n.s.—non-significant difference.

The analysis of IAA status in cotyledons of plants grown under 25 °C revealed that the content of free IAA reached about 21% of the total IAA in invasive lupine and about 14% in non-invasive lupine. The content of this transportable IAA was about twice higher in cotyledons of invasive plants than in non-invasive plants (Figure 2).

The major part of total IAA in cotyledons of both tested lupine species was in a bound form. The content of these reversible low-molecular mass complexes (IAA-esters and amides) reached at least 70% of the total IAA content (Figure 2). The content of IAA-amides and IAA-esters was higher in cotyledons of invasive lupine; however, the proportions of these IAA conjugates were equivalent in both lupine species. The amount of IAA conjugates was 30% higher in cotyledons of invasive lupine. This led us to predict that seedlings of invasive lupine would be provided with a higher amount of free IAA. A negligible and almost similar part of total IAA (9–13%) was identified as IAA catabolites (irreversibly degraded) in cotyledons of both tested lupine species (Figure 2).

**Figure 3:**
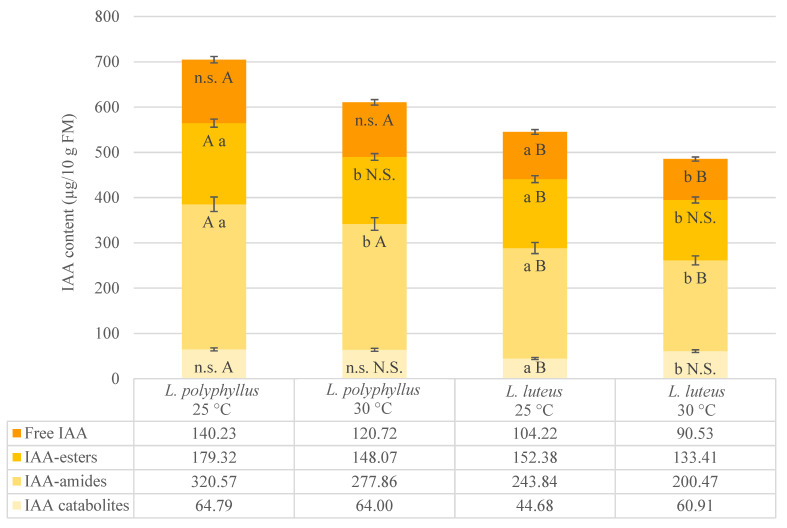
IAA content in hypocotyls of seedlings grown at 25 °C and 30 °C. Vertical bars represent the total IAA content. Sub-bars depict the content of IAA compounds. Values are mean ± SD of three experiments with five replicates in each. Different uppercase letters indicate significant difference (*p* < 0.05) between mean values of two lupine species grown under the same temperature; N.S.—non-significant difference. Significant difference between mean values of each lupine species at 25 °C and 30 °C is marked with different lowercase letters; n.s.—non-significant difference.

Meanwhile, the content of free IAA was 26% higher in hypocotyls of invasive lupine than in non-invasive lupine (Figure 3). The amount of IAA conjugates, especially of IAA-amides, was also greater in hypocotyls of *L. polyphyllus* (by about 23%). The content of IAA catabolites was 31% higher in hypocotyls of invasive lupine (Figure 3).

Two IAA-amides—indole-3-acetyl-L-aspartic acid (IAA-Asp) and indole-3-acetyl-L-glutamic acid (IAA-Glu)—were identified in cotyledons and hypocotyls of non-invasive lupine, and three—IAA-Asp, IAA-Glu and indole-3-acetyl-L-alanine (IAA-Ala)—were identified in cotyledons and hypocotyls of invasive lupine (Table 2 and Table 3). Both lupine species contained one IAA-ester-type conjugate—IAA complex with glucose (IAA-Glc)—and one IAA catabolite—2-oxindole-3-acetic acid (Ox-IAA) (Table 2 and Table 3). The results on the 30 °C temperature effect on the total IAA content in tested plants reveal that the total IAA content was 20% higher in invasive than non-invasive lupine (Figure 2 and Figure 3).

The content of free IAA decreased by 13% in cotyledons and hypocotyls of *L. polyphyllus* under elevated temperature (Figure 2 and Figure 3, Table 2 and Table 3). The same temperature regime had a similar effect on the free IAA level in hypocotyls and a more substantial effect (reduced twice) in cotyledons of *L. luteus*. The content of IAA-esters (IAA-Glc and IAA-Glu) under 30 °C decreased in both species. The decrease in IAA-Glc content was greater in the invasive plants (Figure 2, Table 2). The major decrease in IAA-Glu content (up to 18%) was in cotyledons of both species. The IAA-amide composition changed unevenly in both lupines under 30 °C (Table 2 and Table 3). A major, more than 20% decrease was detected in IAA-Asp content in tissues of both tested lupine organs, especially in cotyledons of *L. polyphyllus* (by 38%). It must be noted that the IAA-Ala complex was detected in seedlings of invasive lupine only (Table 2 and Table 3). Its content in cotyledons was lower than in hypocotyls, and the 30 °C temperature affected it differently. IAA-Ala content increased by 11% (70.84 ± 5.54 μg/10 g of fresh mass at 25 °C and 78.68 ± 1.81 μg/10 g of fresh mass at 30 °C) in hypocotyls and decreased by 7% in cotyledons under simulated warming conditions.

No significant changes under simulated 5 °C warming were detected in the content of IAA catabolite Ox-IAA in invasive lupine. However, it increased by 18% in cotyledons and by 36% in hypocotyls of non-invasive lupine (Table 2 and Table 3).

### 2.3. Ethylene Production under 25 °C and 30 °C

**Figure 4:**
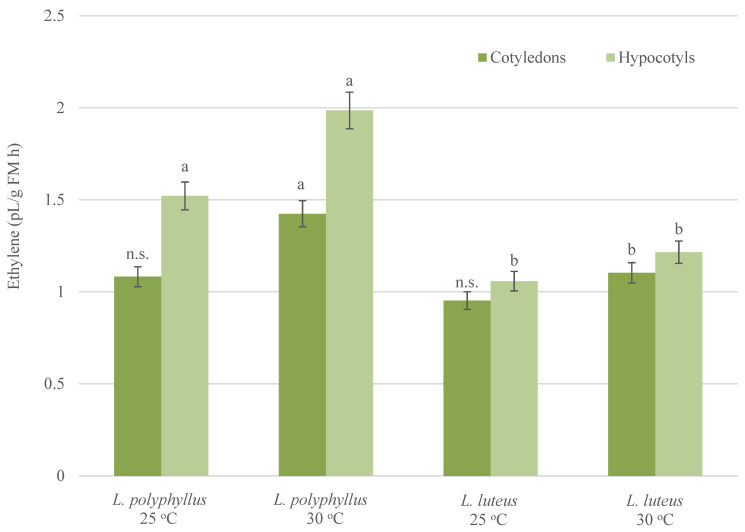
Ethylene production in seedlings grown at 25 °C and 30 °C. Values are mean ± SD of three experiments with five replicates in each. Different letters indicate significant difference (*p* < 0.05) between two lupine species grown under the same temperature; n.s.—non-significant difference.

Comparative analysis of ethylene production in hypocotyls of both lupine species under 25 °C showed significant differences. Ethylene emission in hypocotyls of invasive lupine was 29% higher than that in non-invasive lupine (Figure 4). However, no significant differences were found in ethylene emission from cotyledons of both investigated lupine species.

The elevated temperature significantly (up to 30%) increased ethylene production in both tested organs of invasive lupine. The production of ethylene in hypocotyls and cotyledons of non-invasive lupine was less intensive. This research reveals statistically significant differences in ethylene emission in tissues of invasive and non-invasive lupines under simulated warming conditions. Emission values in hypocotyls and cotyledons of invasive lupine were found to be higher by 38% and 21%, respectively, as compared to non-invasive lupine tissues (Figure 4).

## 3. Discussion

*L. polyphyllus* originates from North America [38,41]. It is one of the most common alien plants in Europe. It has been planted as a fodder crop and as an ornamental plant and is now widely naturalized. It is one of the seven most aggressive invasive plant species in Lithuania [46]. *L. polyphyllus* changes meadow and sand communities and eliminates uncompetitive native plants [47]. *L. luteus* originates from the Mediterranean region of Southern Europe. It is cultivated as a fodder and cover crop and is non-invasive in temperate regions.

Invasive and non-invasive species differ in many traits [48,49]. In general, invasive alien plants have broad environmental tolerance and are usually characterized by fast growth [7,8,48]. It is thought that elevated temperature may increase invasion risk by accelerating physiological processes and growth by increasing the competitive ability of invasive species [5,6,7,49,50]. On the other hand, there is evidence that climate warming may cause declines in populations of invasive plants [2,5,51]. Our data show that the temperature of 30 °C is not optimal for the growth of invasive lupine in the early stages of development as well as for non-invasive lupine as compared to the growth at 25 °C (Table 1, Figure 1). Furthermore, the data of the current study show that the seed germination of invasive lupine was 5% lower under 30 °C (Table 1). On the other hand, the seed germination of non-invasive lupine remained the same at 30 °C. This agrees with the data on *L. polyphyllus* germination at high summer temperatures in the studies of other authors [52]. Therefore, the tendency to climate warming seems to be not beneficial for the germination and growth of invasive *L. polyphyllus*.

Numerous studies have shown the role of phytohormones in plant reactions to environmental changes. The important regulators of adaptive plant growth responses to environmental stresses are IAA and ethylene [38,53]. Their content in invasive and non-invasive lupines was analyzed in the current study. Analysis of the IAA state in cotyledons and hypocotyls of both lupine species showed that IAA homeostasis was maintained through both key IAA metabolic pathways—reversible conjugation and catabolism through non-decarboxylative oxidation (Figure 2 and Figure 3). This has also been shown in early phases of growth in other plants [33,54,55,56,57]. Previous studies have shown that ox-IAA is a major primary IAA catabolite in higher plants, which has low biological activity and is important in the regulation of IAA homeostasis and stress response mechanisms [32,43,58]. Data of the current study show that 8–19% of the IAA content in hypocotyls and cotyledons of both tested lupine species can be catabolized through the oxidative catabolic pathway.

The data of this experiment show a decrease in the total amount of IAA in cotyledons and hypocotyls of both tested lupine species grown at 30 °C, compared to plants grown at 25 °C (Figure 2 and Figure 3). The studies of IAA turnover in cotyledons at the early phases of growth of invasive and non-invasive lupines showed that the 5 °C temperature increase (from 25 up to 30 °C) affected the hydrolysis of IAA reversible complexes (IAA-amides and IAA-esters) in both lupine species (Figure 2 and Figure 3). Earlier, it was shown that IAA-Asp, IAA-Glu and IAA-Glc are common in higher plants, and various physiological roles in growing parts are attributed to them [17,54,59]. However, the contribution of these conjugates in particular developmental pathways as well as in specific invasive and non-invasive plant responses to temperature changes (e.g., simulated climate warming) is unknown. Some studies have presented evidence that IAA conjugates may be involved in abiotic stress tolerance [17,60]. The mutant cell line of henban with impaired IAA-Asp biosynthesis dies at 33 °C to which the wild type is resistant [61]. Our results show a significant decrease in the IAA-Asp amount at 30 °C in both lupine species, especially in cotyledons of invasive lupine (by 38.57%). This could indicate that the 5 °C warming effect induced temperature stress in *L. polyphyllus*. Nevertheless, the IAA-Ala complex has been detected in several plants, though its role has been poorly investigated [54,58]. There are few data suggesting that IAA-Ala could be related to plant growth inhibition [17,62,63]. Similar results were obtained in the current study with *L. polyphyllus* seedlings at 30 °C. The IAA-Ala complex was found only in invasive lupine. Moreover, the proportion in the content of IAA-amides was greater at 30 °C compared to 25 °C (Table 2 and Table 3).

Our results show that the content of free IAA and IAA conjugates (except IAA catabolites) was higher in *L. polyphyllus* than that of *L. luteus* at both temperatures (Figure 2). The content of free IAA under 30 °C was about three-fold higher in cotyledons and 33% higher in hypocotyls of invasive lupine than in non-invasive lupine. The higher level of free IAA in hypocotyls of invasive lupine seedlings may have resulted from the maintenance of the level of IAA in cotyledons (Table 2 and Table 3). The higher amount of IAA can be transported from cell to cell, interact with specific receptors, moderate IAA inducible gene expression and participate in growth and development processes [27,33,64]. The role of IAA in the post-germination growth period was obvious in both lupine species under simulated warming conditions (Table 1, Table 2 and Table 3). This is in agreement with the data on the role of IAA under changing environmental conditions, including global warming, obtained by other authors [14,15,17,27,53].

Studies have shown that the plant hormone ethylene participates in numerous aspects of plant development. The content of ethylene can be modified by biotic and abiotic factors [18,37,38]. The production of ethylene increases in response to temperature stress and enables plants to reach a high level of plasticity and to adapt to environmental changes [16,38,40]. These studies have shown that invasive plants are more adaptable to changing environmental conditions (e.g., increased temperature). They are more sensitive to environmental stress and reduce growth processes in a timely manner. In our study, the growth reduction of hypocotyls of invasive lupine at 30 °C could be triggered by elevated ethylene production. These growth adaptations enable plants to minimize the risk of heat damage and enhance evaporative leaf cooling for optimal plant growth [65]. Results of the current study show that elevated temperature has an impact on ethylene emission in cotyledons and hypocotyls at early phases of development. It was increased in both lupine species under warming conditions. However, the rise in ethylene production in invasive lupine was higher (Figure 4).

The diversity of the ethylene function is thought to be achieved in combination with other phytohormones, e.g., IAA [16,35,66,67]. A significant alteration of ethylene emission in cotyledons of invasive lupine may be related to significant changes in the utilization of IAA resources and a higher level of free IAA. An increase in ethylene emission and a decrease in IAA content in seedlings of invasive lupine at 30 °C were observed in this study. These results show the possible link between phytohormones IAA and ethylene and the manifestation of invasiveness under simulated climate warming.

The obtained data show the possible role of phytohormones IAA and ethylene in adaptive responses of invasive plants to simulated warming at the early developmental phase. It was determined that the level of IAA conjugates (especially of IAA-amides) and transportable IAA, as well as ethylene emission, was higher in invasive than in non-invasive lupine. The decreased level of transportable IAA and IAA conjugates and increased ethylene emission were detected under elevated temperature in seedlings of both species. These changes coincided with the slower growth of *L. polyphyllus*. However, the growth of *L. luteus* was stimulated under elevated temperature conditions. Additionally, the IAA-amide IAA-Ala was found in invasive lupine only. The data of the current study show that the hormonal traits of invasive and non-invasive lupines were altered by elevated temperature at the early seedling phase. It can be suggested that the invasive properties of these plants may be changed by global warming scenarios.

## 4. Materials and Methods

### 4.1. Plant Material and Treatments

**Figure A1:**
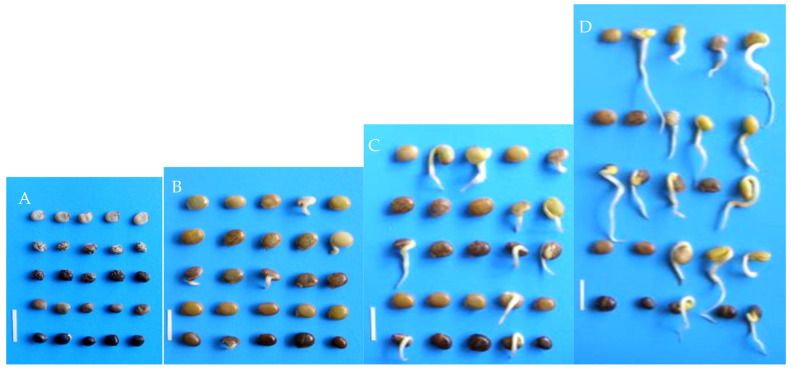
Development of *L. polyphyllus* seedlings under 25 °C temperature: 0 h (**A**), 24 h (**B**), 48 h (**C**) and 72 h (**D**). Scale bar, 1 cm.

Seeds of two lupine species (*L. polyphyllus* Lindl. and *L. luteus* L.) were used in the study. One hundred seeds of each lupine species (with five replications) were soaked in distilled water and grown in climate chambers (Climacell, Czech Republic) at 90% relative humidity in the dark at two different temperatures: at 25 °C (optimal temperature for lupine) and 30 °C (simulated climate warming temperature) [49]. The germination of soaked seeds was monitored (Figure A1), and germinated seeds were counted. The germination percentage was calculated.

Following germination, seedlings were grown for four days under previous conditions at 25 °C and 30 °C.

Cotyledons and hypocotyls of four-day-old invasive and non-invasive lupines (Figure 1) were separated and weighed after being washed with sterile distilled water for determination of fresh mass and assays.

### 4.2. Indole-3-acetic Acid Assay

#### 4.2.1. Extraction and Hydrolysis of IAA

IAA compounds were extracted from the samples of plant material by grinding with 80% methanol, containing 1 mg/L antioxidant butylated hydroxytoluene at a ratio of 1:10 (*w/v*), using a porcelain mortar with a pestle and incubated for 16 h at 4 °C in the dark using a Multi-Pulse Vortexer device (Glass-Col, Terre Haute, IN, USA). The extracts were separated from the residue by filtration through 0.2 μm pore size membrane filters (Whatman, Maidstone, UK), and after removing phenolic compounds by poly(vinylpolypyrrolidone) (PVP40) (Sigma, Neustadt, Germany) at concentration of 0.5%, they were concentrated using a vacuum evaporator (IKA RV-10 Basic, Germany) until dry. Following ether and ethylacetate extractions of the IAA compounds, extracts were purified from peptides and small biomolecules (molecular weight > 700–1500) on a Sephadex G-10 or G-15 column. Indole compounds were detected using specific coloring Salkowski and Ehrlich reagents. The alkaline hydrolysis method was applied for IAA-ester and IAA-amide quantification according to free IAA release following IAA-ester complex hydrolysis at 1 N NaOH 30 °C for 30 min and IAA-amide complex hydrolysis at 7 N NaOH 100 °C for 1 h [32,44,54,57,68].

#### 4.2.2. Chromatographic Isolation and Quantitative Estimation of IAA

Thin-layer chromatography (TLC) and high-performance liquid chromatography (HPLC) were used to separate the individual indole compounds [48,54,68]. The IAA compounds were separated on Alugram SIL G/UV 24 TLC plates (Macherey-Nagel, Düren, Germany), detected under UV light λ = 254 nm and identified by comparing their RF values with synthetic standards. The absorption spectra (λ range: from 220 to 320 nm) of IAA and other indoles were generally identified using a UV–Vis spectrophotometer, Specord 210 PLUS (Analytik Jena GmbH, Jena, Germany). Amounts of indole compounds were calculated (µg/10 g of fresh mass) using the calibration curve.

**Figure 5:**
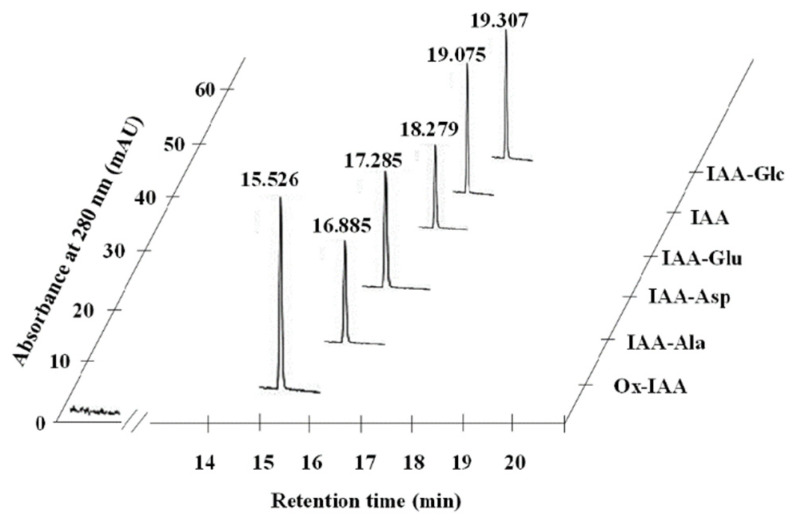
Separation of standards (1 pmol) of IAA and IAA conjugates under HPLC analysis. Ox-IAA—2-oxindole-3-acetic acid; IAA-Ala—indole-3-acetyl-L-alanine; IAA-Asp—indole-3-acetyl-L-aspartic acid; IAA-Glu—indole-3-acetyl-L-glutamic acid; IAA—indole-3-acetic acid; IAA-Glc—IAA complex with glucose.

Final identification of IAA and its metabolites was performed by HPLC analysis according to the procedures described by Kowalczyk and Sandberg [69], using a Shimadzu PROMINENCE LC-20 series system (Shimadzu Technologies, Kyoto, Japan). The samples were separated in a reversed phase column, YMC-PackPro18 (YMC CO, Japan), with a particle diameter of 3 μm. The linear gradient of eluent A (methanol) was from 1 to 95% (*v/v*) in eluent B (water acidified by 1% acetic acid (*v/v*)), at a flow rate of 0.6 mL/min, a time span of over 45 min and an oven temperature of 30 °C. IAA and its metabolites were identified by co-elution with authentic standards. IAA and IAA metabolites (five IAA-amides, one IAA-ester and three IAA catabolites) were used as standards to evaluate the composition and quantity of IAA compounds. (Figure 5). The standard compounds were purchased from OlChemIm Ltd. (Olomouc, Czech Republic) and Sigma-Aldrich (Germany).

### 4.3. Ethylene Assay

Ethylene production was determined in freshly harvested samples of hypocotyls and cotyledons. Samples of a known mass were placed in 30 mL glass vials sealed with a rubber stopper [70]. After 24 h of incubation in the dark at the same temperatures in which plants were grown (at 25 °C and 30 °C), 1 mL of head gas was sampled from each vial, and the ethylene content was measured using a FOCUS GC (Thermo Fischer Scientific, Italy) gas chromatographer, equipped with a flame ionization detector and a stainless-steel matrix 80/100 column, PROPAC R (Sigma-Aldrich, USA). The carrier gas was helium. The temperatures of the column, injector and detector were 90 °C, 110 °C and 150 °C, respectively. Ethylene contents were expressed in picolitres evolved per gram of tissue per hour (pL/g h). Ethylene standard (Alltech, Germany) was used to quantify the content of ethylene in the samples.

### 4.4. Statistical Analysis

The data presented are mean values ± standard deviation (SD) of three experiments with five replicates in each. Germination test was performed in three independent experiments with five replications. Each replicate included one hundred seeds of each lupine species. All analytical data are expressed on a fresh mass basis. The data were statistically analyzed using analysis of variance (ANOVA) and tested for significant mean differences (*p* < 0.05) using Tukey’s test. Statistical analyses were performed with SPSS Statistics v. 17.0 (SPSS Inc., Chicago, IL, USA) software.

## 5. Conclusions

The obtained data show the role of phytohormones IAA and ethylene in the adaptive response of invasive L. *polyphyllus* and non-invasive *L. luteus* to simulated warming at the early developmental phase.

The germination rate of *L. polyphyllus* decreased under elevated 30 °C temperature; nevertheless, the rate of germination of *L. luteus* remained the same comparing to the optimal 25 °C temperature.

The simulated warming resulted in decreased growth of *L. polyphyllus* at the early seedling phase as compared to *L. luteus*.

A higher amount of total IAA, especially of IAA-amides, and transportable IAA, as well as a higher amount of ethylene emission, was characteristic for *L. polyphyllus* under both temperatures in comparison to *L. luteus*.

Additionally, the IAA-amide IAA-Ala was found in invasive *L. polyphyllus* only.

A higher supply of IAA in cotyledons of *L. polyphyllus* seedlings at the early phases of development was observed due to the intensive hydrolysis of IAA-amides.

The decreased level of transportable free IAA and IAA conjugates and increased ethylene emission were detected under elevated temperature in seedlings of both species. These changes coincided with the slower growth of *L. polyphyllus*.

The data of the current study show that the hormonal traits of both tested lupine species were altered by the elevated temperature at the early seedling phase.

## Figures and Tables

**Table 1:** Effect of temperatures of 25 °C and 30 °C on germination and growth parameters of four-day-old seedlings of two lupine species.

Species	Temperature, °C	Germination, %	Fresh Mass, g		
			Cotyledons	Hypocotyls	Roots
*L. polyphyllus*	25	36.65 ± 1.03 a	0.906 ± 0.15 n.s.	1.04 ± 0.16 a	0.30 ± 0.01 a
	30	31.28 ± 1.18 b	0.825 ± 0.19 n.s.	0.61 ± 0.11 b	0.15 ± 0.05 b
*L. luteus*	25	39.82 ± 1.36 n.s.	2.229 ± 0.30 n.s.	1.12 ± 0.21 a	0.21 ± 0.06 n.s.
	30	39.24 ± 1.54 n.s.	2.271 ± 0.29 n.s.	1.46 ± 0.22 b	0.27 ± 0.06 n.s.

Values are mean ± SD of three experiments with five replicates in each. Different lowercase letters indicate significant difference (*p* < 0.05) between mean values at 25 °C and 30 °C for each lupine species; n.s.—non-significant difference.

**Table 2:** The comparison of IAA content in cotyledons of four-day-old seedlings of two lupine species grown at 25 °C versus 30 °C.

IAA Form		Changes in Amount (%)	
		*L. polyphyllus*	*L. luteus*
Free IAA	IAA	↓ 13.10 ± 0.85 *	↓ 50.96 ± 3.46 *
IAA-esters	IAA-Glc	↓ 12.70 ± 1.23 *	↓ 8.40 ± 2.03 *
IAA-amides	IAA-Glu	↓ 18.20 ± 1.16 *	↓ 18.33 ± 1.23 *
	IAA-Asp	↓ 38.57 ± 2.65 *	↓ 22.98 ± 2.60 *
	IAA-Ala	↓ 6.88 ± 0.29 *	Non-detected
IAA catabolites	Ox-IAA	↑ 9.03 ± 0.28	↑ 18.17 ± 1.88 *

Values are mean ± SD of three experiments with five replicates in each. ↑ and ↓—increase and decrease in content, respectively. *—significant difference (*p* < 0.05) between mean values at 25 °C and 30 °C for each lupine species.

**Table 3:** The comparison of IAA content in hypocotyls of four-day-old seedlings of two lupine species grown at 25 °C versus 30 °C.

IAA Form		Changes in Amount (%)	
		*L. polyphyllus*	*L. luteus*
Free IAA	IAA	↓ 13.90 ± 0.90	↓ 13.13 ± 0.46 *
IAA-esters	IAA-Glc	↓ 17.29 ± 1.15 *	↓ 12.42 ± 0.33 *
IAA-amides	IAA-Glu	↓ 12.55 ± 0.23 *	↓ 16.46 ± 0.57 *
	IAA-Asp	↓ 27.23 ± 1.85 *	↓ 21.17 ± 1.46 *
	IAA-Ala	↑ 11.10 ± 0.44 *	Non-detected
IAA catabolites	Ox-IAA	↓ 2.77 ± 0.30	↑ 36.32 ± 2.98 *

Values are mean ± SD of three experiments with five replicates in each. ↑ and ↓—increase and decrease in content, respectively. *—significant difference (*p* < 0.05) between mean values at 25 °C and 30 °C for each lupine species.

## Data Availability

The data supporting reported results can be found in scientific reports of the Laboratory of Plant Physiology of Institute of Botany of Nature Research Centre, where archived datasets generated during the study are included.
